# Differential Effects of Human SP-A1 and SP-A2 on the BAL Proteome and Signaling Pathways in Response to *Klebsiella pneumoniae* and Ozone Exposure

**DOI:** 10.3389/fimmu.2019.00561

**Published:** 2019-03-26

**Authors:** Guirong Wang, Todd M. Umstead, Sanmei Hu, Anatoly N. Mikerov, David S. Phelps, Joanna Floros

**Affiliations:** ^1^Department of Pediatrics, Center for Host defense, Inflammation, and Lung Disease (CHILD) Research, The Pennsylvania State University College of Medicine, Hershey, PA, United States; ^2^Department of Surgery, SUNY Upstate Medical University, Syracuse, NY, United States; ^3^Department of Obstetrics and Gynecology, The Pennsylvania State University College of Medicine, Hershey, PA, United States

**Keywords:** humanized transgenic mice, innate immunity, oxidative stress, proteomic profile, surfactant protein A (SP-A), signaling pathway

## Abstract

Surfactant protein A (SP-A) plays critical roles in host defense, regulation of inflammation and surfactant metabolism in the lung. The human SP-A locus consists of two functional genes, *SFTPA1* and *SFTPA2* encoding surfactant proteins SP-A1 and SP-A2, respectively. Structural and functional differences exist between SP-A1 and SP-A2 *in vitro* and *in vivo*. Ozone is a major air pollutant with a negative impact on many biological processes. In this study we used humanized transgenic (hTG) SP-A1 and SP-A2 mice, and SP-A KO mice to study *in vivo* effects of SP-A1 and SP-A2 on the bronchoalveolar lavage (BAL) proteomic profile and associated signaling pathways in response to ozone or filtered air (FA) exposure and *Klebsiella pneumoniae* infection. The BAL samples were harvested 24 h after ozone (2 ppm for 3 h) or FA exposure and infection and analyzed by two-dimensional difference gel electrophoresis (2D-DIGE) and MALDI-ToF/ToF. We found: that (1) Ozone exposure, but not infection, is a major factor for increases in total BAL protein content. (2) A total of 36 proteins were identified, accounting for 89.62% of the BAL proteins resolved by the 2D-DIGE system. (3) The number of proteins in which levels were altered more than 25% following infection and FA exposure was: SP-A2 > SP-A1 > KO for male mice, and SP-A2 ≈ SP-A1 > KO for female mice. (4) The number of proteins with more than 25% increase/decrease after ozone exposure and infection was: SP-A2 > SP-A1 ≈ KO, with the majority being increases in male mice and decreases in female mice. (5) Eleven out of the 36 proteins, including annexin A5, glutathione S-transferase A4, SP-A1/SP-A2, and 14-3-3 zeta protein, exhibited significant differences among SP-A genotypes. The acute phase response (APR) that includes the NF-kB signaling pathway plays a critical role, followed by Nrf2-mediated oxidative response, and others. These associated with SP-A genotype, sex, and ozone-induced oxidative stress in response to infection. We concluded that human SP-A2 and SP-A1 exhibit differential genotype-and sex-dependent innate immune responses to microbial pathogens and/or ozone-induced oxidative stress by modulating proteomic patterns and signaling pathways in the lung.

## Introduction

Surfactant protein A (SP-A) is a surfactant-associated protein that plays an important role in innate host defense, regulation of inflammation, and surfactant-related physiology in lung (1, 2). SP-A is a member of the C-type lectin (collectin) protein family. Each monomer consists of four domains, N-terminal region, collagen-like domain, neck domain, and carbohydrate-recognition domain (CRD). Native SP-A from bronchoalveolar lavage (BAL) is thought to consist primarily of octadecamers with six SP-A trimers. The literature from SP-A knockout (KO) mouse research revealed that SP-A is a critical factor for host defense (3–7), surfactant metabolism (8, 9), surfactant large-aggregate structure and tubular myelin formation (10–12) in the lung, and survival after infection (13, 14).

The human SP-A locus consists of two functional genes, *SFTPA1* and *SFTPA2* encoding SP-A1 and SP-A2, respectively, plus a pseudogene and is located on chromosome 10q22-23 (15, 16). Each of the functional genes has been characterized and several genetic variants for each gene have been identified. The SP-A1 variants (6A, 6A^2^, 6A^3^, 6A^4^) and the SP-A2 variants (1A, 1A^0^, 1A^1^, 1A^2^, 1A^3^, 1A^5^) are the most frequently found variants in the population (17, 18). Human SP-A is expressed in alveolar epithelial type II cells (19), as well as in other tissues (20–22). SP-A2 expression has been observed in tracheal and bronchial submucosal gland cells in the lung (23, 24). SP-A1 or SP-A2 genetic variants have been associated with several human pulmonary diseases (25). Structural and functional differences between SP-A1 and SP-A2 and among *in vitro* expressed SP-A genetic variants have been observed using several different approaches (26–37). SP-A2 for the most part appeared to exhibit higher activity in terms of its ability to enhance phagocytosis by alveolar macrophages (29, 31–33), enhance cytokine production by a macrophage-like cell line (27, 30, 38), and inhibit surfactant secretion by alveolar epithelial type II cells (28). Moreover, SP-A1 and SP-A2 differentially enhance aggregation of LPS and phospholipid monolayer formation (26, 35). In a study of humanized transgenic (hTG) mice the function of SP-A1 and SP-A2 was shown to have diverged in terms of tubular myelin (TM) formation, an extracellular form of pulmonary surfactant (39). Recently, *in vivo* functional differences between SP-A1 and SP-A2 genetic variants were observed in the regulation of alveolar macrophage actin cytoskeleton (40), the alveolar macrophage proteome (37, 41), the alveolar macrophage microRNAome (42), pulmonary mechanics (43) and bacterial-induced mortality (44). However, in a recent study SP-A1 was found to more efficiently affect surfactant structural organization compared to SP-A2 (36).

Ozone is one of the major air pollutants that can have a negative impact on a variety of biological processes including inflammation, increased airway reactivity, and an increased susceptibility to lung infection in humans (45–49). Ozone exposure can affect innate immunity, epithelial integrity, impair phagocytosis, and compromise mucociliary clearance (45, 50), and therefore can modulate risk for many respiratory diseases including asthma (47). Furthermore, a large population study demonstrated a significant increase in the risk of death from respiratory causes, with an increase of ozone concentration (51). Differences among individuals in ozone-induced symptoms have also been observed and polymorphisms in genes related to oxidative stress may underlie these differences Ozone-exposure has shown to have a negative impact on SP-A function (52). Differential effects on the function of SP-A1 and SP-A2 *in vitro* (28, 30, 33, 38) and *in vivo* (42, 52) have been observed.

In the present study we used gel-based proteomic analysis as a discovery tool to study potential molecular mechanisms, Specifically we used the 2D-DIGE proteomic approach along with MALDI-ToF/ToF to analyze global changes of proteins in lung BAL fluid to study the BAL proteome in hTG mice, each carrying an SP-A1 or SP-A2 variant, in response to *K. pneumoniae* infection and in the presence or absence of ozone.

## Materials and Methods

### Animals

We have generated and characterized hTG SP-A1 (6A^4^) and SP-A2 (1A^3^) mice on the SP-A−/− (KO) C57BL/6 genetic background (39). These mice express human SP-A1 or SP-A2 and secrete it into BAL fluid. In this study, we used these hTG SP-A1 and SP-A2 mice, as well as SP-A KO C57BL/6 mice from the same litters as controls. The mice used in these experiments were 10–12 weeks old and ~25 g in body weight. The mice were bred and maintained under pathogen-free conditions and fed rodent chow and tap water *ad libitum* in the animal core facility. This study was approved by the Institutional Animal Care and Use Committee at the Pennsylvania State University College of Medicine.

### Bacterial Strain

*Klebsiella pneumoniae* bacteria (ATCC 43816) were purchased from the American Tissue Culture Collection (Rockville, MD). Bacteria were cultured in 250 ml flasks with 50 ml of 3% tryptic soy broth (TSB) for 18 h at 37°C with shaking at 100 rpm. The bacterial culture was diluted in TSB to obtain an OD_660_ of 0.4 and then 200 μl of the diluted bacterial suspension was added to 50 ml of TSB medium and shaken for 3 h to reach mid-log phase of growth (OD_660_ ~0.4, corresponding to ~2 × 10^8^ CFU/ml). Bacteria were placed on ice to stop their growth and then serially diluted in PBS to obtain ~9 × 10^3^ CFU/ml. Mice were infected by the intratracheal instillation of 50 μl of the suspension with ~450 CFU/mouse.

### Experimental Design and Mouse Model

A total of forty-eight 10–12 week old mice (8 male and 8 female hTG SP-A1mice, 8 male and 8 female hTG SP-A2 mice, and 8 male and 8 female SP-A KO mice) were divided into 12 groups with 4 animals per group (Table 1).

**Table 1:** Experimental design.

**Mouse genotype**	**Male**		**Female**	
	**FA + infection**	**O_**3**_ + infection**	**FA + infection**	**O_**3**_ + infection**
SP-A1 (6A^4^)	SP-A1M_FA	SP-A1M_O_3_	SP-A1F_FA	SP-A1F_O_3_
SP-A2 (1A^3^)	SP-A2M_FA	SP-A2M_O_3_	SP-A2F_FA	SP-A2F_O_3_
SP-A KO	KOM_FA	KOM_O_3_	KOF_FA	KOF_O_3_

*Four mice were used in each of the 12 groups*.

Mice were exposed to either 2 parts/million (ppm) ozone or to filtered air (FA) for 3 h as described previously (13, 53). In brief, four mice were put into a glass exposure vessel with stainless steel wire mesh lids and then placed in a closed glass exposure chamber. Exposures to FA were conducted in parallel at room temperature and 50% humidity using the ozone exposure system as described previously (50, 53, 54). After FA or ozone exposure, the mice were immediately infected with *K. pneumoniae* bacteria. This was done by anesthetizing the mice, surgically exposing the trachea, and instilling 50 μl of bacterial suspension. The mice were sacrificed 24 h after infection by anesthetizing them with an intramuscular injection of a 40 μl mixture of Ketamine/HCl a (Ketaset, Fort Dodge Animal Health, IO) and Xylazine (XYLA-JECT, Phoenix Pharmaceuticals Inc., St. Joseph, MO) and exsanguination. The lungs were lavaged with normal saline.

### Cell Counts and Total Protein Assessment in BAL

BAL was obtained by instilling 0.6 ml of saline into the lungs three times through a tracheal cannula using a volume equal to 80% of lung vital capacity (for a total of 1.5 ml). Total BAL fluid recovery was ~90% of the instilled volume and did not differ significantly between experimental groups. The BAL was centrifuged (150 × g, 10 min, 4°C) and the cell pellet was resuspended in saline (0.9% sodium chloride). Total cell counts were performed using a hemocytometer and cytocentrifuge (Cytospin) preparations were used to obtain differential cell counts. The cell-free BAL supernatant was frozen at −80°C for subsequent proteomic studies. Total protein concentrations in BAL were determined using the Bio-Rad protein assay (Bio-Rad, Hercules, CA).

### TCA/Acetone Precipitation

One volume of ice cold 100% trichloroacetic acid (TCA) was added to four volumes of BAL, mixed and incubated overnight at 4°C. Following overnight incubation, samples were centrifuged (15,000 × g, 15 min, 4°C) and the protein pellets washed with 250 μl of chilled acetone, centrifuged again, and resuspended in a minimum volume of standard cell lysis buffer (30 mM Tris-HCl, 2 M thiourea, 7 M urea, 4% CHAPS, pH 8.5). The concentration of protein was adjusted to 2 mg/ml for CyDye labeling.

### 2D-DIGE Labeling (Minimal Labeling) and Electrophoresis for 2D-DIGE

Information about the 2D-DIGE is provided in a form that complies with the most recent version <http://www.psidev.info/sites/default/files/MIAPE_GE_1_4.pdf> of Minimum Information About a Proteomics Experiment—Gel Electrophoresis (MIAPE-GE) standards developed by the Human Proteome Organization Proteomics Standards Initiative (55) (Supplemental File 1). 2D-DIGE has been widely used to study the proteomic profiles (37, 41, 50, 54, 56–59). The methods of 2D-DIGE labeling and electrophoresis for 2D-DIGE used in this study have been described in previous publications (37, 41, 50, 54, 56, 59, 60).

For preparative (picking) gels an aliquot of 500 μg of sample was diluted with an equal volume of 2X sample buffer (2 M thiourea, 7 M urea, 2% IPG buffer (pH 4–7) and 1.2% DeStreak reagent) and then brought up to a volume of 450 μl with rehydration buffer (DeStreak™ Rehydration Solution and 0.5% IPG buffer (pH 4–7). Proteins were focused using the following voltages and times: 14 h at 0 V (passive rehydration); 6 h at 30 V (active rehydration); 3 h at 300 V (step and hold); 3 h at 600 V (gradient); 3 h at 1,000 V (gradient); 3 h at 8,000 V (gradient); 4 h at 8,000 V (step and hold). Each of the strips was equilibrated as described above and applied to a 10% polyacrylamide preparative picking gel (26 cm-w × 20 cm-h × 1 mm thick). After the completion of electrophoresis the preparative picking gels were stained with Deep Purple Total Protein Stain (GE, Healthcare) as described (50, 56, 60).

### Gel Scanning and Image Analysis

Information about the acquisition and processing of data from the 2D-DIGE studies are provided in the form that complies with the most recent version of the guidelines established for Minimum Information about a Proteomics Experiment—Gel Informatics (MIAPE-GI) developed by the Human Proteome Organization Proteomics Standards Initiative http://www.psidev.info/sites/default/files/MIAPE_GE_1_4.pdf (Supplemental File 2). 2D-gels were scanned as described previously (50, 56, 60).

Gel images were imported into the Progenesis SameSpots v3.0 (Nonlinear Dynamics, Durham, NC) for analysis as described previously (50, 60). Gel alignment was conducted automatically and then checked manually to ensure correct alignment. A reference gel with minimum distortion and streaks was then selected from the Cy2 gels. Detection and matching of protein spots across all the gels was conducted automatically, then manually checked and edited to ensure correct matching, merging, and splitting of spots. All the included spots were transported to Progenesis PG240 module of the Progenesis SameSpots v3.0 software. Quantization of spots was accomplished by comparing the ratio of each Cy3 and Cy5 value to the values obtained from the normalization pool/Cy2 channel present on each gel.

### Protein Identification by Mass Spectrometry

To identify specific proteins we collected spots from the preparative picking gel. We digested the gel pieces with trypsin and then eluted the digested peptides from the gel and analyzed them by MALDI-ToF/ToF as described previously (50, 56, 60). Peptides were analyzed by MALDI-ToF/ToF in the Mass Spectrometry Core at the Penn State University College of Medicine. A total of 5 μl of ZipTip cleaned samples (1 μl at a time) was applied onto a 384-well MALDI plate (Opti-TOF™ 384 Well Insert, Applied Biosystems, Foster City, CA) and then 0.7 μl of 2 mg/ml ACH cinnamic acid in 73:27 (acetonitrile:water) was then spotted on each well containing peptide. All 13 calibration wells on the MALDI plate were spotted with (1:12 diluted) 4700 calibrant. Autolytic trypsin peptides were also used to internally calibrate the spectra to an accuracy of 20 ppm. Peptides were then analyzed by MALDI-ToF/ToF mass spectrometry using a 4800 Proteomics Analyzer (Applied Biosystems), calibrated with Applied Biosystems 4700 Proteomics Calibration Mix. Using GPS Explorer 3.0 software (Applied Biosystems), the MS and MS/MS data were submitted to a MASCOT (v2.0.00) search engine for identification. The NCBI nonredundant database with the *Mus musculus* taxonomy and a concatenated, reversed “decoy” version were used for the searches with a mass accuracy of 50 ppm, 1 missed trypsin cleavage, fixed carbamidomethylation of cysteine residues and variable oxidation of methionine residues. A protein was considered identified if the MASCOT confidence interval was >95th percentile and those proteins with a MASCOT confidence interval <95% were excluded from the subsequent analyses.

The PANTHER database and the scientific literature were used to assign molecular function and biological process to each identified protein. As in our previous publications (37, 41, 50, 56, 59–61), we assigned the identified proteins to three broad functional groups in this study: (1) host defense proteins (DEF); (2) proteins involved in redox metabolism and balance (RED); (3) proteins involved in protein metabolism and modification (PMM). It should be noted that some proteins are involved in multiple functional groups in this study. In addition, the Ingenuity Pathway Analysis program (Ingenuity Systems, Redwood City, CA) was used to analyze the identified proteins and their changes between different types of mice in order to gain additional insight into the functional significance and the signaling pathway(s) involved.

### Determination of NRF2 Levels in the Lungs of Ozone-Exposed Mice

In a separate study, C57BL/6J mice were exposed to 2 PPM of ozone for either 1, 2, or 3 h with 0, 1, 2, or 3 h of recovery time after ozone. Lungs were then harvested and nuclear and cytoplasmic extracts were prepared using Thermo Scientific NE-PER Nuclear and Cytoplasmic Extraction Reagents and following the manufacturer's instructions. Ten micrograms of each extract were then separated by SDS-PAGE and the proteins transferred to nitrocellulose for detection and quantitation of NRF2 levels by Western ECL and densitometry.

### Statistical Analysis

Initial statistical analysis was performed in Progenesis SameSpots v3.0 by Student's *t*-test to confirm the level of significance among various groups. For identified proteins having multiple isoforms, the normalized volumes of all isoforms of a given protein were added together and statistical analysis was repeated on the totals. In the following analysis, all the data were expressed as mean ± SEM and analyzed by one-way analysis of variance or student's *t* test using the standard program software SigmaStat (version 3.5, SPSS). Differences were considered statistically significant at *p* < 0.05.

## Results

As stated in the section of Materials and Methods (Table 1), all mice in this study were infected with *K. pneumoniae*. Three types of mice used are: (1) hTG SP-A2 (1A^3^); (2) hTG SP-A1 (6A^4^) mice; (3) SP-A KO mice. Equal numbers of males and females were used in this study. Mice were sacrificed 24 h after bacterial infection.

### Cell Counts and Total Protein Concentrations in the BAL

Total cell and differential cell counts in the BAL of each group with varying genotypes, sex, and exposure conditions are listed in Table 2. The data indicate significant differences between FA-exposed and ozone-exposed KO or hTG SP-A2 mice of both sexes. Typically there was a two- to three-fold increase in the ozone-exposed mice. Differences were less pronounced and not significantly different between FA- and ozone-exposed hTG SP-A1 mice of both sexes. Much of the increase in all markers was likely due to an influx of neutrophils, ranging from 14.4 to 37.8% in FA-exposed mice, and from 63.8 to 79.3% in ozone-exposed animals (Table 2). While some of this increase is due to the effect of ozone (53), most of it is probably due to the *K. pneumoniae* infection (56). There were no significant differences in the percentages of neutrophils or macrophages between male and female mice of each genotype and under both exposure conditions.

**Table 2:** Total cells and its composition from mouse BAL.

**Mouse genotype/sex/treatment**	**Cell counts (x10^**4**^)**	**% of Macrophage**		**% of Neutraphil**	
	**Mean ±*SD***	**Mean**	***SD***	**Mean**	***SD***
SP-A1_M_FA	14.62 ± 6.65	62.2	29.1	37.8	29.1
SP-A1_M_O3	23.31 ± 5.5	36.2	30.9	63.8	30.9
SP-A1_F_FA	15.43 ± 15.5	75.1	26.2	24.5	26.5
SP-A1_F_O3	18.75 ± 5.3	31.8	11.4	67.8	11.3
SP-A2_M_FA	6.81 ± 2.9	57.0	45.6	14.4	15.0
SP-A2_M_O3	21.43 ± 9.8	25.3	19.0	74.7	19.0
SP-A2_F_FA	8.18 ± 3.1	69.8	21.7	30.2	21.7
SP-A2_F_O3	21.43 ± 5.1	26.1	11.2	73.8	11.2
KO_M_FA	10.87 ± 4.6	77.3	30.6	22.7	30.6
KO_M_O3	31.87 ± 7.1	26.5	5.6	73.5	5.6
KO_F_FA	9.31 ± 3.4	80.1	10.3	19.9	10.3
KO_F_O3	31.56 ± 15.6	20.8	16.8	79.3	16.8

**Figure 1:**
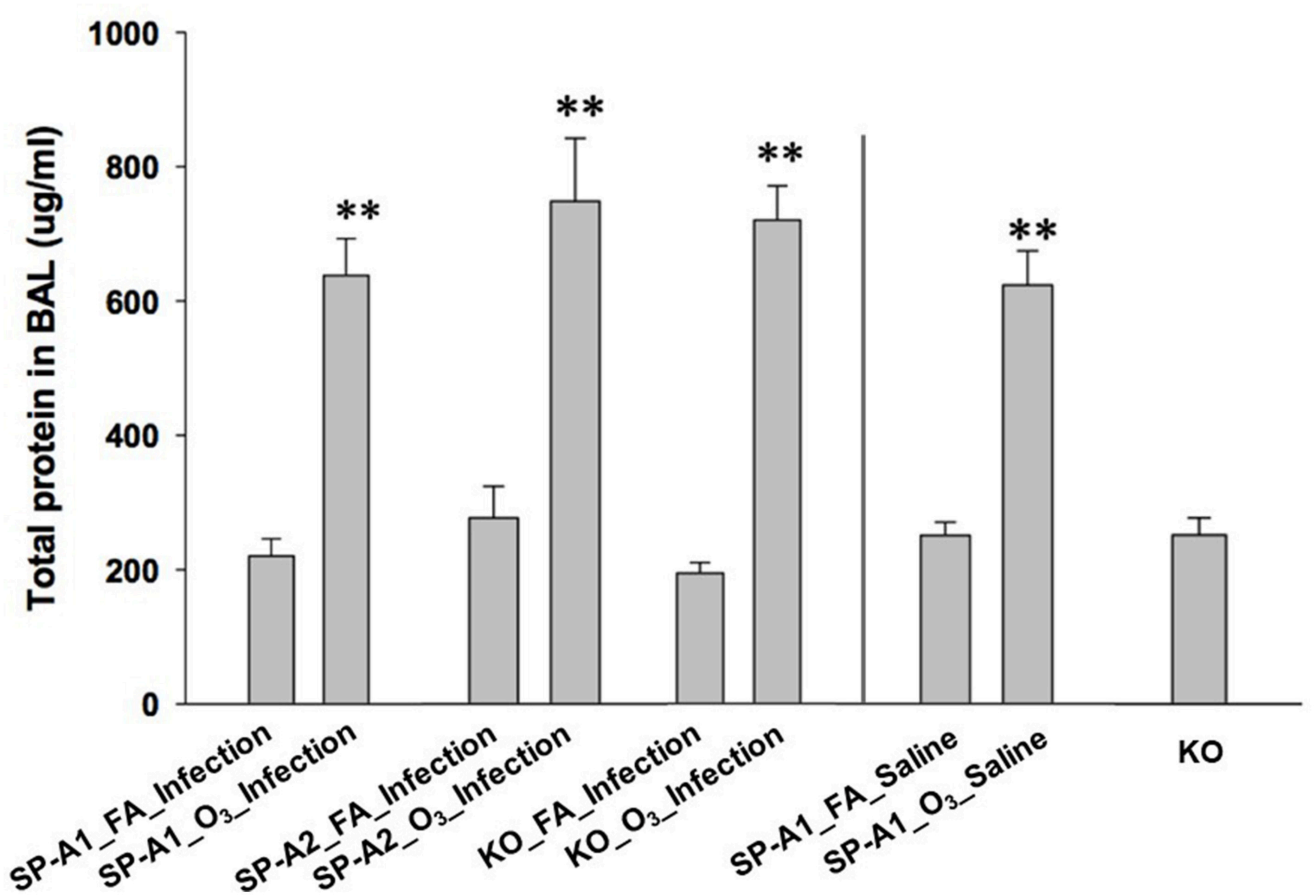
Comparison of total protein concentration in the BAL fluid from exposed and infected mice. Three genotypes of mice (hTG SP-A1 and SP-A2 mice and SP-A KO mice) were exposed to ozone (3 ppm) or filtered air for 3 h and then intratracheally infected with 50 μl of bacterial solution (containing about 450 CFU of *K. pneumoniae*, control with 50 μl of saline). After 24 h of infection the mice were sacrificed and the lungs were subjected to BAL. Total protein concentration was determined in the supernatant of BAL. The results indicate that the total protein level of ozone-exposed mice, regardless of bacterial infection or saline control was significantly higher than that of FA-exposed mice (^**^p < 0.01). No significant difference is found among FA-exposed mouse groups regardless of infection or saline control, or among ozone-exposed mouse groups regardless of infection or saline control. In addition, no significant differences in total protein level in the BAL fluid are observed between KO background control and FA-exposed mice regardless of infection or saline control (The experimental groups and controls were separated by a vertical line).

We also determined the total protein concentration in the BAL of each group. The data from male and female mice of each genotype showed no remarkable differences, therefore, the data from male and female mice of each group were combined in this analysis. The results (Figure 1) indicated that total protein concentration of each ozone-exposed group was significantly higher (approximately three times) than that of the corresponding FA-exposed group (*p* < 0.01). These levels, and the ozone-induced increases, are consistent with observations we have published previously (53) indicating that these are largely a consequence of the ozone exposure rather than the infection. There were no significant differences among the three types of mice of FA-exposed groups, or among the ozone-exposed groups. To better understand the effect of ozone-induced increase of total proteins in BAL, three additional groups, which were not used for other analyses of this study, were used as controls. These were hTG SP-A1 mice with exposure to either ozone (SP-A1_O_3__Saline) or FA (SP-A1_FA_Saline), in which 50 μl of saline were instilled instead of the bacterial suspension, as well as SP-A KO mice without any treatment. These results demonstrate that ozone exposure of mice was the major factor influencing the total protein concentration of the BAL under the present experimental conditions.

### Protein Identification of BAL by 2D-DIGE Analysis of hTG and KO Mice

**Figure 2:**
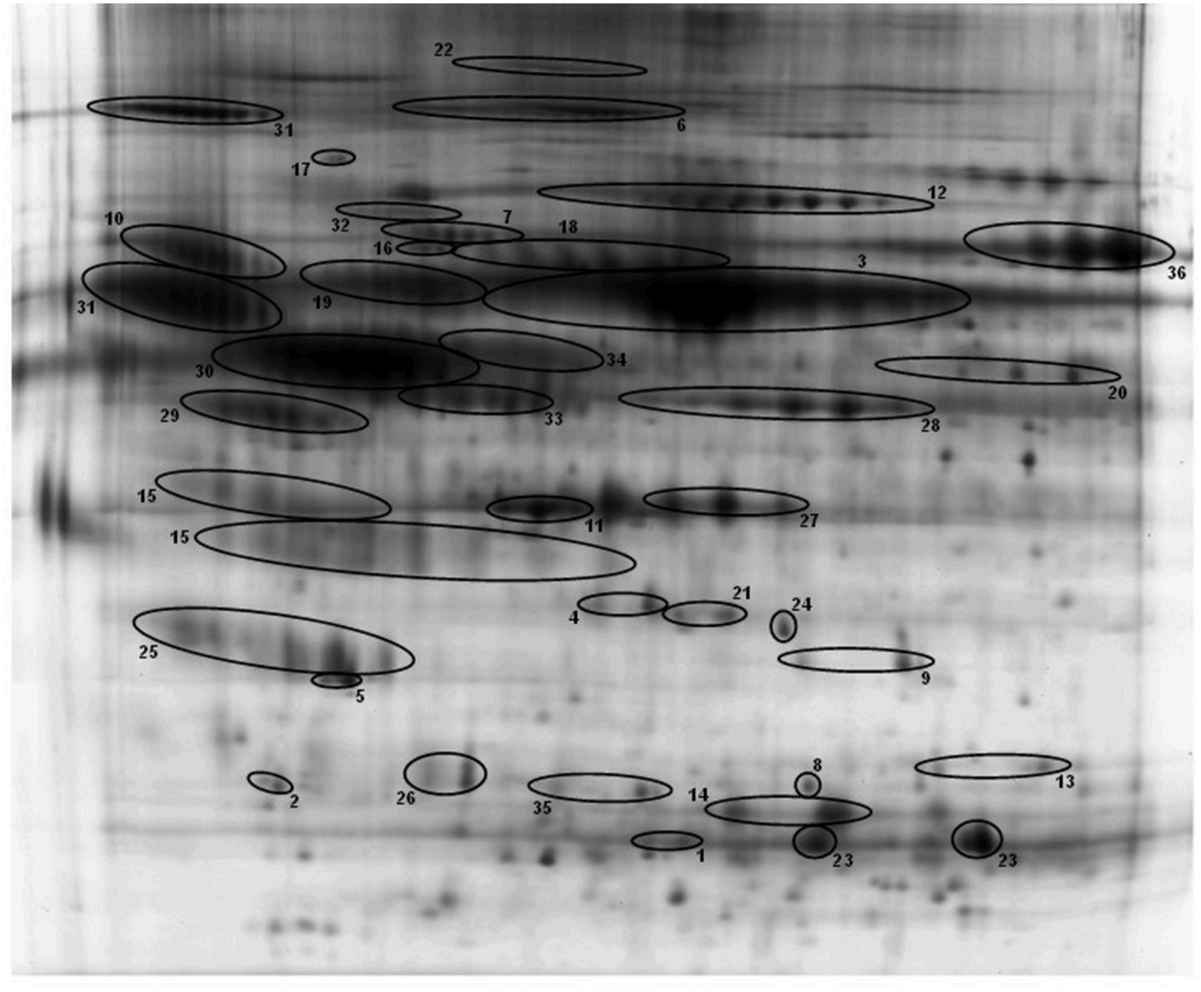
A representative reference gel with identified proteins. Protein spots identified by MALDI-ToF/ToF are circled and numbered, and the names of all the proteins and the corresponding numbers are given in Table 3. Many proteins have multiple isoforms in the 2-D gel and all isoforms of these proteins are identified and confirmed by MALDI-ToF/ToF.

BAL samples of the 12 experimental groups were subjected to 2D-DIGE and analyzed with Progenesis SameSpots. For 2D-DIGE analysis, BAL samples were labeled with Cy3 or Cy5 dye. A normalization pool consisting of equal aliquots of all samples was labeled with Cy2. Samples were separated by isoelectric focusing (pH 4–7) and then 10% PAGE-gel electrophoresis (2-D) as described in the Materials and Methods. A total of 36 proteins from 201 spots were identified by MALDI-ToF/ToF in this study (Table 3). The 36 identified proteins accounted for 89.62% of BAL proteins resolved in our gel system. Figure 2 shows a reference gel in which the 36 identified proteins are circled and numbered. In the case of proteins consisting of multiple isoforms, positive identification by MALDI-ToF/TOF was made on each isoform and statistical analysis was done on the sum of all isoforms. All identified proteins are shown in Table 3.

**Table 3:** List of identified proteins.

**No**.	**Protein ID**	**Gene symbol**	**Accession No**.	**Functional group**
1	(Similar to) Glutathione S-transferase A1	Gsta1	P13745	RED, DEF
2	14-3-3 zeta protein	Ywhaz	P63101	DEF, RED, PMM
3	Albumin	Alb1	P07724	RED
4	Annexin A3	Anxa3	Q3UCL0	DEF
5	Annexin A5	Anxa5	P48036	DEF
6	Ceruloplasmin	Cp	Q61147	RED
7	Complement C5	C5	P06684	DEF
8	Complement component 3	C3	Q80XP1	DEF
9	Cytosolic malate dehydrogenase	Mdh1	P14152	RED
10	Esterase 1	Es1	P23953	DEF
11	Gamma-actin	Actg1	P63260	PMM
12	Gelsolin	Gsn	P13020	DEF, RED, PMM
13	Glutathione S transferase, omega 1	Gsto1	O09131	DEF, RED
14	Glutathione S-transferase A4	Gsta4	P24472	DEF, RED
15	Haptoglobin	Hp	Q60574	DEF, RED
16	Heat shock protein 5	Hspa5	P20029	DEF, PMM
17	Heat shock protein, 1A	Hsp90aa1	Q80Y52	DEF, PMM
18	Hemopexin	Hpxn	Q8K1U6	RED
19	Kininogen 1	Kng1	O08677	DEF, PMM
20	Kpnb1 protein	Kpnb1	Q99KM9	PMM
21	Lactate dehydrogenase 2	Ldhb	P16125	RED, DEF
22	Murinoglobulin-1	Mug1	P28665	PMM, DEF
23	Peroxiredoxin 6	Prdx6	Q6GT24	RED, DEF
24	Pregnancy zone protein	Pzp	Q61838	PMM, DEF
25	Pulmonary surfactant-associated protein A (SP-A)	sftpa1	P35242	DEF, RED
26	Rho GDP dissociation inhibitor (GDI) alpha	Arhgdia	Q99PT1	PMM
27	SEC 14-like 3	Sec14l3	Q5SQ27	PMM
28	Selenium binding protein	Selenbp1	P17563	DEF, RED
29	Serine protease inhibitor, A1a	Serpina1a	P07758	DEF, PMM
30	Serine protease inhibitor, A1c	Serpina1c	Q00896	PMM
31	Serine protease inhibitor, A3k	Serpina3k	P07759	PMM
32	Serine protease inhibitor, A1e	Serpina1e	Q00898	PMM
33	Serine protease inhibitor, A1	Serpina1	P26595	DEF, PMM
34	Serine protease inhibitor (antithrombin III), C1	Serpinc1	P32261	PMM
35	Serum amyloid P-component	Apcs	P12246	DEF, PMM
36	Transferrin	Trf	Q921I1	PMM, RED, DEF

To gain insight into the changes in protein levels, the identified proteins (*n* = 36) were assigned into three broad functional groups as described previously (37, 41, 50, 54, 56, 59–61). One group contained proteins (*n* = 24) involved in defense and immunity functions (DEF), including proteins playing roles in host defense, regulation of inflammatory processes, and other immune processes. The second group consisted of proteins (*n* = 15) playing a role in the regulation of redox balance in the lung (RED). These proteins are involved in the generation of reactive oxygen and nitrogen species (RONS), neutralization of RONS, or are proteins that bind molecules such as iron, copper, and heme. The third group consisted of proteins (*n* = 20) that we broadly categorized as being involved in protein metabolism and modification (PMM), including a number of proteases and antiproteases, several chaperones, proteins involved in molecular signal, and transportation processes (Table 3). It should be noted that many of the proteins studied have been assigned into more than one of the three groups because these proteins play roles in multiple biological processes. To perform 2D-DIGE, the same amount of protein from each sample was loaded on gels. However, because total BAL protein concentration was about 3 times higher in ozone-exposed mice than in FA-exposed mice, in order to have the same amount of protein from both FA- and ozone-exposed samples on gels we loaded a much smaller fraction of the total amount of BAL from the ozone-exposed samples on each gel. To compensate for this we corrected all values for normalized volumes of all spots so that they represented the quantity of each protein spot in the total amount of BAL. These corrected normalized volumes, representing a constant proportion of the total BAL in both ozone-exposed and FA-exposed mice, were used for all subsequent analyses.

### Comparisons of BAL Proteomic Profiles of SP-A Genotypes in Response to FA or Ozone

This study included three experimental variables, e.g., SP-A genotype (SP-A1 or SP-A2), sex (male or female), and exposure (FA or ozone). To compare the influence of each genotype on the BAL proteomic profile 12 group comparisons were studied (Table 1S). The effect of each genotype in these comparisons was studied by examining the 6 male (first set) and 6 female (second set) comparisons separately. The percent change of each of the 36 identified proteins between two samples was calculated with the following formula. For example, to compare sample A to sample B, the % change = 100 × (the average content of A—the average content of B)/the average content of B. The % change of each of the 36 identified proteins, as well as significant difference between groups (*p* < 0.05) are shown in Table 1S. In addition, according to suggestions from previous publications (62, 63), a 25% protein content change (increase or decrease) was used as a threshold, because this is one of the criteria used for defining acute-phase response proteins. Therefore, the 25% change (increase or decrease) was used as a threshold in the subsequent analysis.

#### Infected Male Mice After Exposure to FA or Ozone

##### Comparison of FA-exposed and infected SP-A1 or SP-A2 vs. KO male mice

Based on Tables 1S, 2S, when compared to KO male mice, out of the 36 identified proteins, 15 proteins (42%) in SP-A1 male mice and 30 proteins (83%) in SP-A2 male mice, exhibited marked increases (% change >25%; pink highlighting). In addition, three proteins (i.e., Annexin A5, Glutathione S-transferase A4, and serum amyloid p-component) in SP-A1 male mice and complement component 3 in SP-A2 male mice were markedly decreased (% change > −25%; green highlighting) compared to KO male mice.

##### Comparison of ozone-exposed and infected SP-A1 or SP-A2 vs. KO male mice

In the comparison between infected SP-A1 and KO male mice and in response to ozone, only haptoglobin (no comparison of SP-A was done due to a lack of SP-A expression in KO mice) showed a pronounced increase of 33.6%, but the 14-3-3 zeta protein and the pregnancy zone protein exhibited a decrease by 31 and 26.1%, respectively (Table 1S). However, with ozone exposure, infected SP-A2 male mice exhibited marked increases in 16 (44%) of the 36 proteins compared with infected KO male mice, with 13 of the 16 proteins being in the host defense and immunity category (DEF) (Tables 1S–3S).

##### Comparison between FA- and ozone-exposed and infected SP-A2 and SP-A1 male mice

The results showed that 18 out of the 36 proteins exhibited increases between infected SP-A2M_FA vs. SP-A1M_FA mice (Table 1S), of which 12 of the 18 proteins were involved in host defense and immunity (Table 2S). With ozone exposure, more than half of the proteins (19 of the 36 proteins) showed increases, but one protein (hemopexin) showed a decrease by 27.7% between infected SP-A2M_O_3_ vs. SP-A1M_ O_3_ mice. Thirteen of the increased proteins were also found in the 18 increased proteins in FA exposure and in the 19 increased proteins in ozone exposure, indicating that the observed changes are primarily due to infection and not to ozone exposure. These findings indicate that the SP-A2 (compared to SP-A1) male mice directly or indirectly regulate levels of many proteins in response to FA and ozone.

#### Infected Female Mice After Exposure to FA or Ozone

Fewer differences in the BAL proteomic profile among SP-A genotypes in infected female mice were observed in response to FA or ozone.

##### Comparison of FA- and ozone-exposed and infected SP-A1 or SP-A2 vs. KO female mice

Of the 35 proteins (excluding SP-A due to its absence in KO mice), 14 (40%) proteins and 13 (37%) proteins in FA-exposed and infected SP-A1 and SP-A2 female mice, respectively, showed profound increases compared to KO female mice (data shown in the second set of Table 1S). However, if exposed to ozone, of the 35 proteins, 4 and 12 proteins in infected SP-A1 and SP-A2 mice, respectively, showed marked decreases, and only two proteins (annexin A5 with increase of 145.3% and glutathione S-transferase A4 with increase of 55.3%) in infected SP-A2 female mice were expressed at higher levels than infected KO female mice. These indicate that the infected SP-A2 female mice are profoundly affected and more responsive to ozone exposure than the infected SP-A1 female mice.

##### Comparison of FA- or ozone exposed and infected SP-A2 and SP-A1 female mice

The results demonstrated that out of the total 36 proteins, 4 (11%) proteins (annexin A5, heat shock protein 1A, Kpnb1 protein, and SP-A) with marked increase and 4 (11%) proteins (glutathione S-transferase A1, 14-3-3 zeta protein, glutathione S-transferase A4, and Sec14-like 3) with marked decrease in infected SP-A2 female mice compared to infected SP-A1 female mice exposed to FA (Tables 1S, 2S). If exposed to ozone, infected SP-A2 female mice showed increases in 3 proteins (annexin A5, glutathione S-transferase A4, and SP-A), and decreases in 5 proteins (complement component 3, heat shock protein 1A, Kpnb1 protein, murinoglobulin-1, and Serpin A1 compared to infected SP-A1 female mice (Tables 1S, 2S).

### Specific Proteins With Significant Differences in SP-A Genotypes

Several of the 36 identified proteins exhibited statistically significant differences in the level of protein expression in several comparisons in SP-A genotypes. It should be noted that comparisons between ozone-exposed to FA-exposed infected mice were excluded because of about two thirds of the 36 proteins were found to be significantly different between ozone-exposed and FA-exposed mice. Below we focus on comparisons of infected males and females, exposed to FA or to ozone.

**Figure 3:**
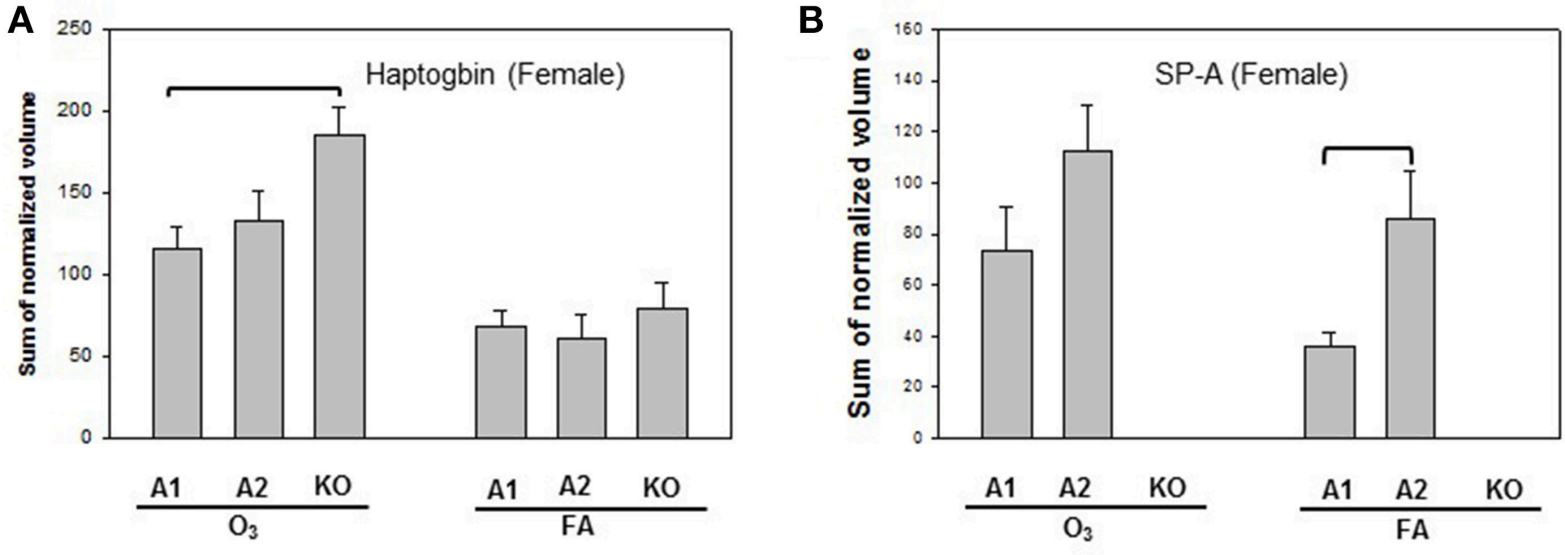
Representative graphs of significant protein changes among genotypes in female mice. Two proteins (haptoglobin and SP-A) exhibit significant differences in comparisons among SP-A genotypes of female mice after exposure to ozone (O_3_) or filtered air (FA). Histograms depicting levels of each specific protein are shown in **(A)** (Haptoglobin) and **(B)** (SP-A). Comparisons between groups that were statistically significant (*p* < 0.05) are indicated with a bracket.

**Figure 4:**
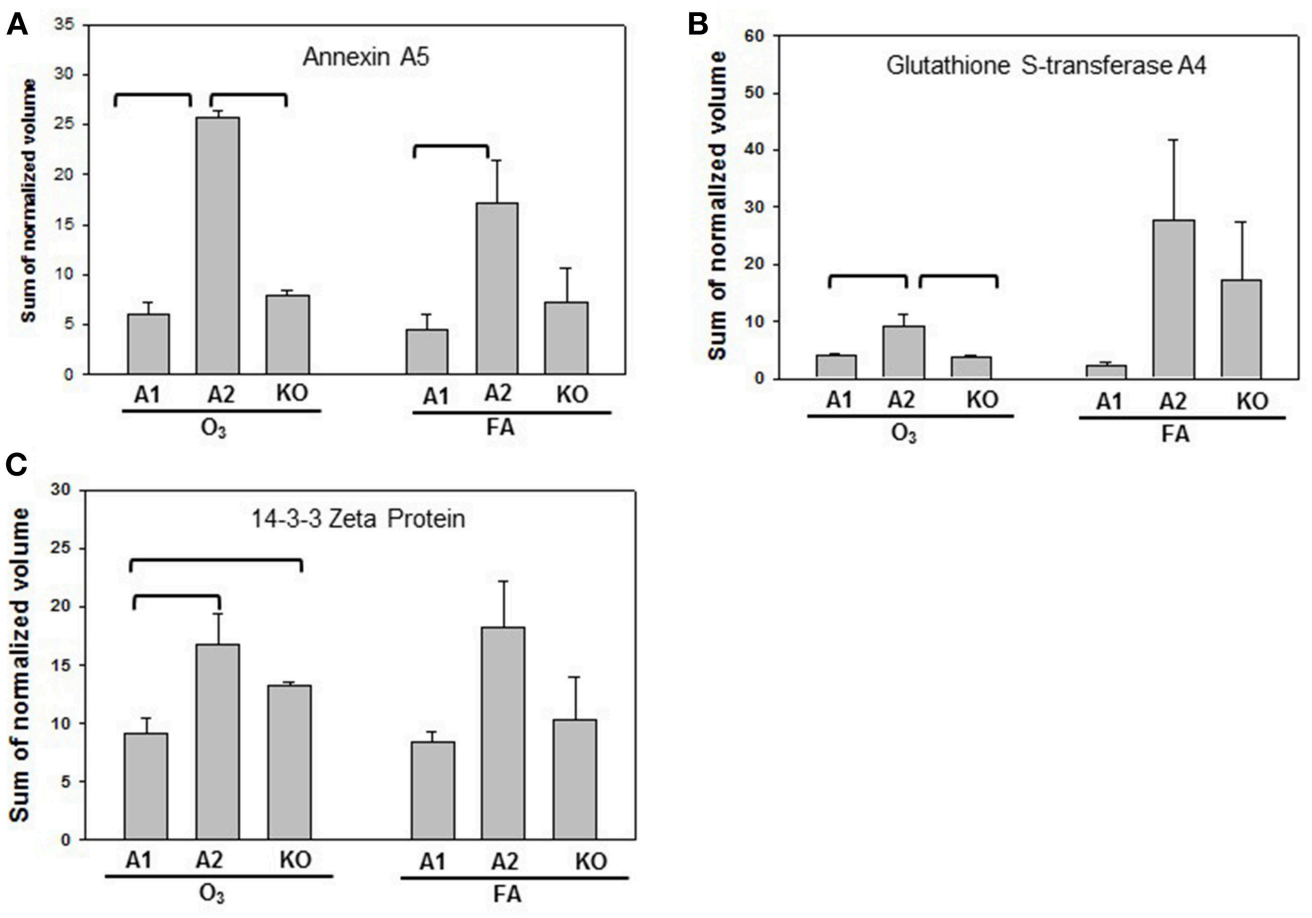
Representative graphs of significant protein changes among genotypes in male mice. The proteins shown exhibit significant differences in at least 2 group comparisons among genotypes of male mice [SP-A1 (A1), SP-A2(A2), KO] after exposure to O_3_ or filtered air (FA) following bacterial infection. Histograms depicting levels of each specific protein includes: **(A)** annexin A5; **(B)** glutathione S-transferase A4; **(C)** 14-3-3 zeta protein. Comparisons between groups that were statistically significant (*p* < 0.05) are indicated with a bracket.

Statistically significant differences were observed primarily in males with only two protein changes in female mice (i.e., SP-A level between SP-A1F_FA and SP-A2F_FA, and haptoglobin level between SP-A1F_O_3_ and KOF_O_3_) (Figure 3). In male mice, three specific proteins exhibited significant differences in at least two of the 6 comparisons (Figure 4). These proteins include annexin A5, 14-3-3 zeta protein, and glutathione S-transferase A4. These are involved in host defense (annexin A5, 14-3-3 zeta protein, and glutathione S-transferase A4), redox balance (glutathione S-transferase A4), and protein metabolism (14-3-3 zeta protein) in lung physiology and pathophysiology (64).

**Figure 5:**
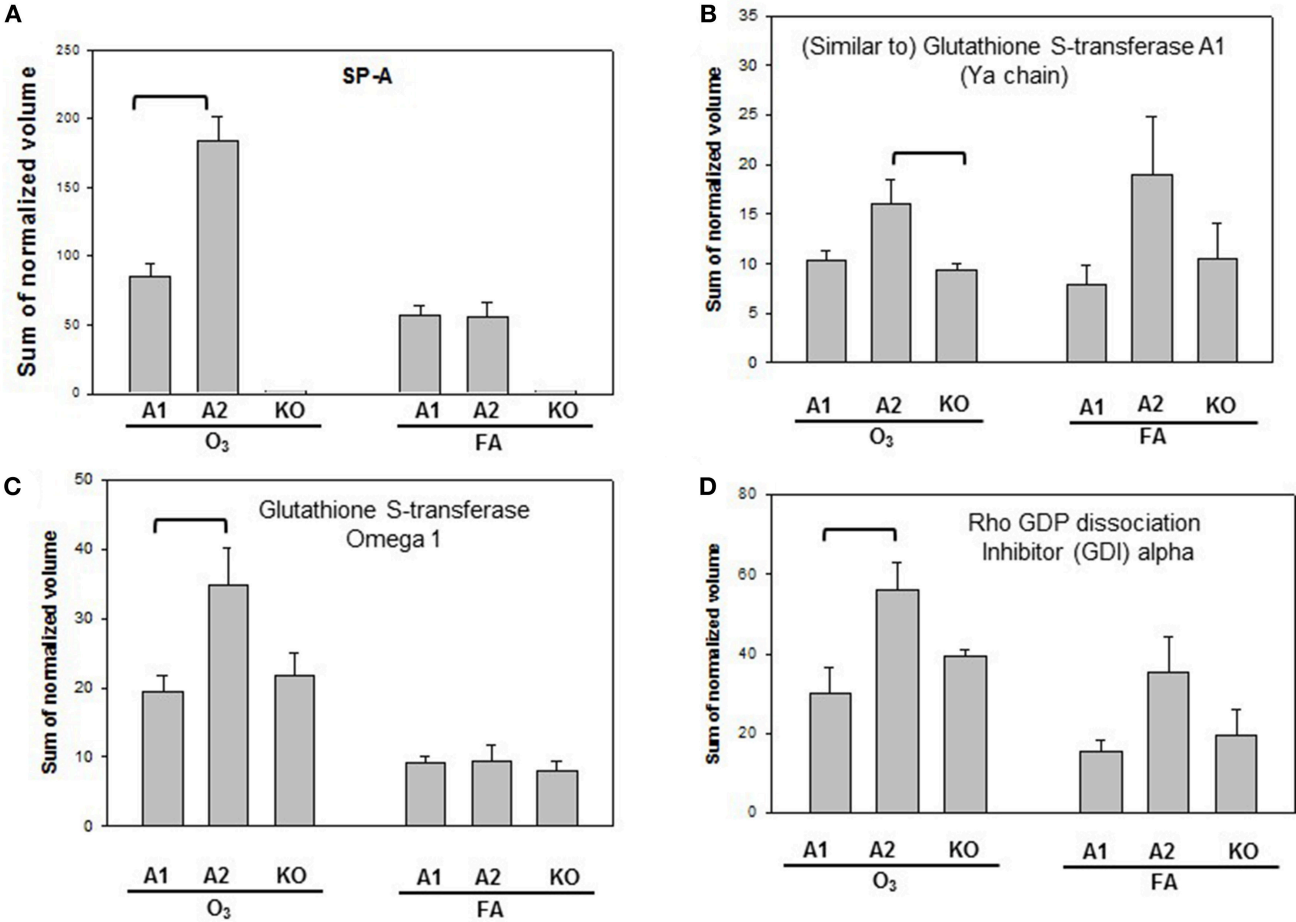
Representative graphs of specific protein changes among genotypes of ozone-exposed male mice. Histograms depict levels of each specific protein, **(A)** SP-A; **(B)** (Similar to) glutathione S-transferase A1; **(C)** glutathione S transferase, omega 1; **(D)** Rho GDP dissociation inhibitor (GDI) alpha. These proteins exhibit significant differences in one group of comparisons of male mice with different genotypes (A1, A2, KO) after exposure to O_3_ and bacterial infection. Comparisons between groups that are statistically significant (*p* < 0.05) are indicated with a bracket.

**Figure 6:**
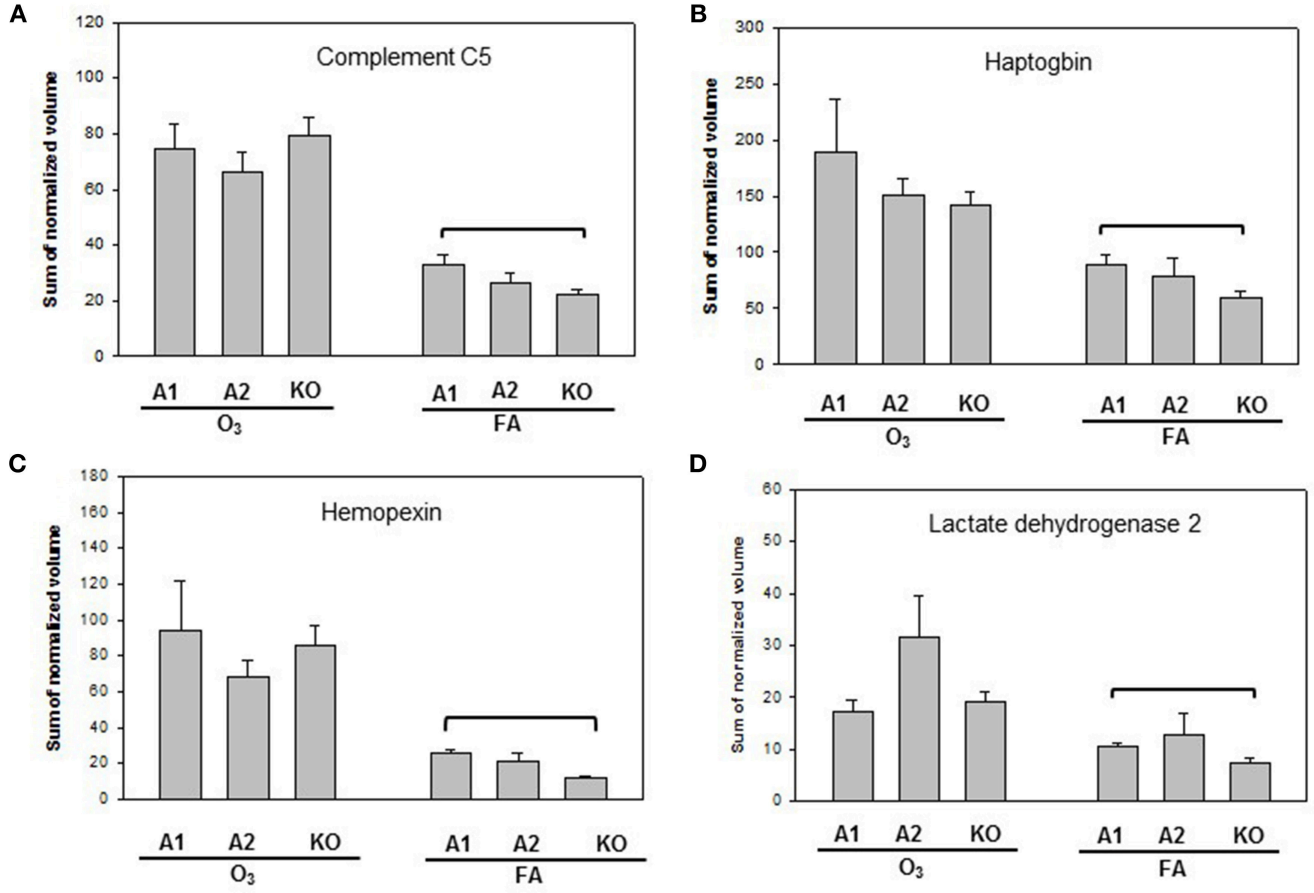
Representative graphs of specific protein changes among genotypes of FA-exposed mice. Histograms depicting levels of each specific protein are included in **(A)** complement C5; **(B)** haptoglobin; **(C)** hemopexin; and **(D)** Lactate dehydrogenase 2. These proteins exhibit significant differences in one of the comparisons of male mice with different genotypes (A1, A2, KO) after exposure to filtered air (FA) and bacterial infection. Comparisons between groups that were statistically significant (*p* < 0.05) are indicated with a bracket.

Proteins with significant differences in male mice after ozone exposure that are found only in one comparison out of 6 comparisons made are shown in Figure 5, and include SP-A, (similar to) glutathione S-transferase A1, glutathione S-transferase, omega 1, and Rho GDP dissociation inhibitor, alpha. As stated in the introduction, SP-A plays a critical role in host defense, regulation of inflammation, homeostasis, and anti-oxidative damage in lung (1, 65). Ozone-exposed SP-A2 mice had higher level of glutathione S-transferase A1 than ozone-exposed KO mice and had higher levels of glutathione S-transferase, omega 1, and Rho GDP dissociation inhibitor, alpha, than ozone-exposed SP-A1 male mice. Thus, the SP-A2 mice in response to ozone not only express a higher level of SP-A protein compared to SP-A1 mice (Figure 5A), but they appear to directly or indirectly regulate/influence the expression of other proteins (also see Figure 4). Of interest, SP-A content in SP-A1 and SP-A2 male mice in response to a single insult (infection) is the same, but in response to a double insult (infection and ozone exposure) the SP-A content in the SP-A2 mice is significantly higher (*p* < 0.05) than that in SP-A1 mice (Figure 5A). On the other hand, in female FA infected SP-A2 mice the SP-A content is significantly increased (*p* < 0.05) by 137.3% compared to SP-A1 female mice, but no differences were observed in SP-A levels between SP-A1 and SP-A2 female mice after ozone exposure (Figure 3B). In addition, four proteins (complement C5, haptoglobin, hemopexin, and lactate dehydrogenase 2) exhibited small but significant changes in FA-exposed SP-A1 mice compared to FA-exposed KO male mice (Figure 6).

### Signaling Pathways Associated With Ozone Exposure and Infection

**Figure 7:**
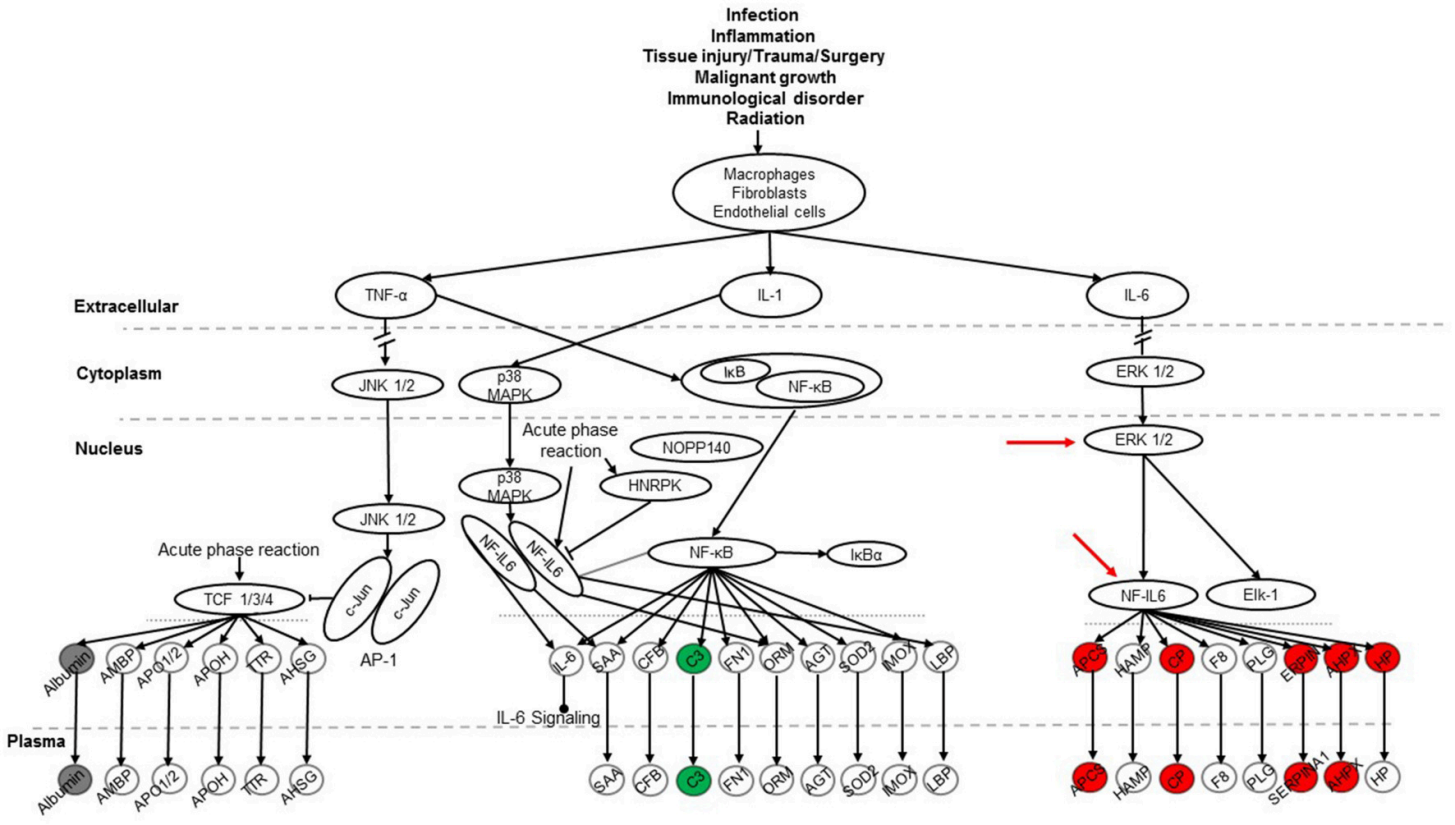
The APR signaling pathway is associated with ozone exposure and bacterial infection. A total of 36 proteins were identified in this study and were used for Ingenuity Pathway Analysis. The canonical “APR signaling” pathway was found to be associated with these proteins (*p* < 4.17E-11). An example of the comparison among SP-A genotypes is shown for the SP-A2M_FA vs. KOM_FA comparison where proteins with increases in (>25%) expression are shown in red, proteins with decreases (>-25%) are shown in green, and proteins with changes <25% are shown in gray. ERK1/2 and NF-IL6 in the pathway are indicated with red arrows.

To explore signaling pathways involved in this experimental model the 36 identified proteins were analyzed with the Ingenuity Pathway Analysis program. The results indicate that 11 proteins (albumin, ceruloplasmin, complement C5, complement component 3, haptoglobin, hemopexin, Serine protease inhibitor A1, Serine protease inhibitor A1c, Serine protease inhibitor A1e, serum amyloid P-component, transferrin) of the 36 proteins are related to the acute phase response (APR) signaling pathway (*p*-value = 4.17E-11), in which most of them were associated with the ERK1/2 and NF-IL6 signaling pathways (Figure 7). Most of the 11 proteins found with a marked change in the present study were members of the NF-IL6 pathway. For example, the comparison between SP-A2M_FA and KOM_FA (shown in Figure 7), revealed that 8 (ceruloplasmin, haptoglobin, hemopexin, serine protease inhibitor A1, serine protease inhibitor A1c, serine protease inhibitor A1e, serum amyloid P-component, transferrin) of the 11 proteins increased more than 25% (marked in red) and one (complement component 3) decreased more than 25% (marked in green); the other two (marked in gray) exhibited <25% change. As shown in Figure 7 the three proteins encoded by serine protease inhibitor A1, serine protease inhibitor A1c, serine protease inhibitor A1e are shown together as “SERPINA1.” The differences in the levels of protein expression of the 11 proteins between SP-A2 and KO male mice provide evidence that the SP-A2 protein could regulate/influence the expression of other proteins that are involved in the APR signaling pathway, specifically in the ERK1/2 and NF-IL6 pathways in this experimental model.

The SP-A1M_FA vs. KOM_FA comparison revealed a similar number of proteins with changed levels (>25%) (8 in the SP-A1 comparison vs. 9 in the SP-A2 comparison described above). Six of the changing proteins; ceruloplasmin, haptoglobin, hemopexin, serine protease inhibitor A1c, serine protease inhibitor A1e, and transferrin were the same between the SP-A1M_FA and SP-A2M_FA, when each of them was compared to KOM_FA. All showed a similar trend (either increase or decrease) with the exception of serum amyloid P-component which was decreased in the SP-A1M_FA and increased in the SP-A2_FA. However, in FA-exposed females of either genotype or in response to ozone a considerably fewer number of proteins in the APR pathway exhibited changes (>25%) for FA: SP-A1 *n* = 1 (albumin with increase); SP-A2 *n* = 1 (haptoglobin with decrease); for ozone: SP-A1 *n* = 1 (complement C5 with decrease); SP-A2 *n* = 3 (all decreases of complement component 3, haptoglobin, and serine protease inhibitor A1c). The results indicate that the role of SP-A genotype in response to bacterial infection and FA or ozone exposure may be through the APR (ERK1/2 and NF-IL6) signaling pathway, especially for males in response to infection and FA exposure.

Furthermore, four additional pathways were identified to be related to the BAL protein changes although only a few proteins were associated with these pathways. These include the Nrf2-mediated oxidative stress response pathway (*p*-value = 3.98E-4) with 4 changed proteins (gamma-actin, glutathione S-transferase A1, glutathione S transferase, omega 1, and glutathione S-transferase A4), the glutathione metabolism pathway (*p*-value = 2.2E-5) with 3 changed proteins (glutathione S-transferase A1, glutathione S transferase, omega 1, and glutathione S-transferase A4), the aryl hydrocarbon receptor signaling pathway (*p*-value = 1.81E-4) with 4 proteins (heat shock protein 1A, glutathione S-transferase A1, glutathione S transferase, omega 1, and glutathione S-transferase A4), and the xenobiotic metabolism signaling pathway (*p*-value = 2.47E-4) with 5 proteins (heat shock protein 5, glutathione S-transferase A1, glutathione S transferase, omega 1, and glutathione S-transferase A4, esterase 1). The three members (glutathione S-transferase A1, glutathione S transferase, omega 1, and glutathione S-transferase A4) of the glutathione S-transferase family were determined to be associated with all the identified pathways. We observed differences in the levels of expression of several members of glutathione S-transferase family in several comparisons among SP-A genotypes, indicating that there may be an important effect of the SP-A genotype on the activation of these in response to bacterial infection and FA/ozone exposure.

### Experimental Confirmation of Nrf2 Signaling Activation

**Figure 8:**
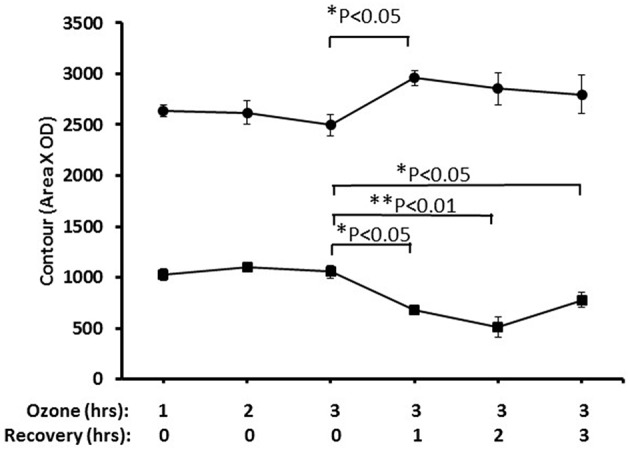
Nuclear and cytoplasmic levels of NRF2 in lung tissue after ozone exposure. Mice were exposed to 2 PPM ozone for 1, 2, or 3 h and then allowed to recover for either 0, 1, 2, or 3 h after exposure. Lungs were then removed, and nuclear and cytoplasmic extracts prepared and run on SDS-PAGE gels. NRF2 levels were quantitated by Western blot ECL and densitometry (• nuclear, ■ cytoplasmic). The results indicated the time-dependent translocation of Nrf2 from cytoplasm to nucleus after ozone exposure (^*^P < 0.05; ^**^P < 0.01).

Nrf2 has been identified as an important pathway in proteomics studies of this study, as well as previous studies (41, 59, 61), where the alveolar macrophage proteome in hTG SP-A1 and SP-A2 mice was studied in response to ozone-induced oxidative stress and/or infection. Here we performed a pilot experiment to confirm its involvement in ozone-induced oxidative stress. Nuclear and cytoplasmic extracts of the lung tissue from treated mice, exposed to ozone (2 ppm) for 1, 2, and 3 h with a post-exposure recovery period of 0, 1, 2, and 4 h were used to study the change of Nrf2 level. Figure 8 shows a time-dependent translocation of Nrf2 to the nucleus following ozone-induced oxidative stress, indicating that Nrf2 does play a role in the system.

## Discussion

SP-A plays critical roles in host defense, regulation of inflammation, and surfactant-related physiology in the lung. Structural and functional differences have been observed between human SP-A1 and SP-A2 proteins expressed *in vitro* by insect or mammalian cells (26–34) and *in vivo, ex vivo*, or *in vitro* (36, 37, 39–44). In the present study we have used hTG SP-A1 and SP-A2 and SP-A KO mice, and studied *in vivo* the effect of human SP-A1 and SP-A2 genotypes on BAL proteomic profile and associated signaling pathways in response to bacterial infection in the presence or absence of ozone.

**Figure 9:**
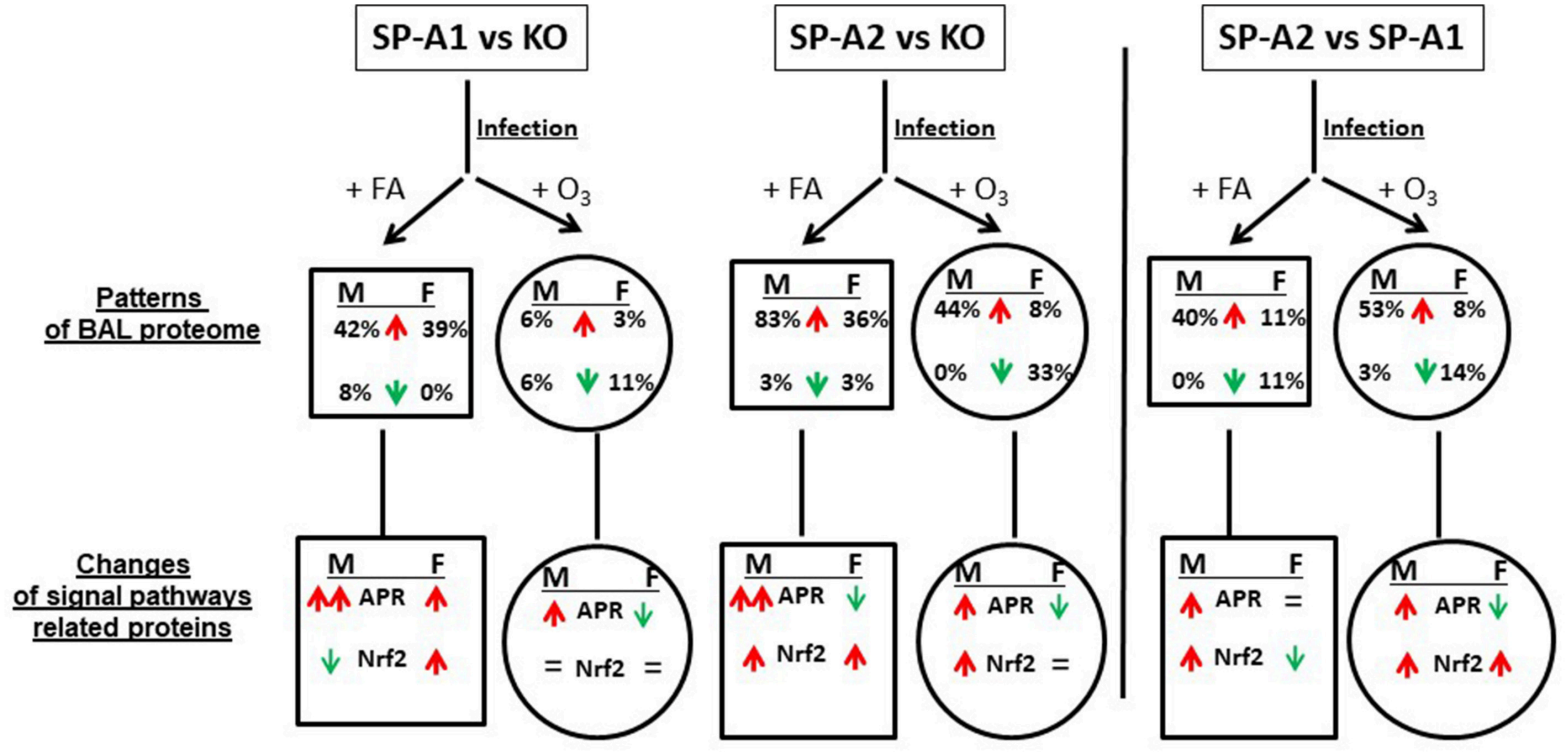
A summary of the BAL proteome and signaling pathways in this study. This figure, following synthesis of all the data, depicts patterns of male (M) and female (F) mice of SP-A1 vs. KO, SP-A2 vs. KO, and SP-A2 vs. SP-A1 after FA or ozone exposure and bacterial infection. An up (↑) arrow (red) and down (↓) arrow (green) indicate increases (>25%) and decreases (>-25%), respectively. The percentage of changed proteins (>25% increase or >-25% decrease) is shown under M and F. In the signaling pathway of related proteins, up (↑) arrows (red) indicate that the number of proteins involved in the signaling pathways with increases (>25%) is more than that of the proteins showing decreases (>-25%). The number in double up (↑↑) arrows (red) is more than in single up (↑) arrows (red). Down (↓) arrows (green) indicates that the number of decreased proteins (>-25%) is more than the number of increased proteins. “=” means no marked changes of proteins (<25%). Based on this model, we found differential effects of SP-A genotypes and sex on the patterns of BAL proteomes and signaling pathways in response to ozone exposure and bacterial infection.

All the data from this study are summarized in Figure 9. This Figure shows that after FA exposure and infection, when compared to KO, the number of proteins with increased level (>25%) are SP-A2 > SP-A1 > KO in male mice, and SP-A2 ≈ SP-A1 > KO in female mice, and after ozone exposure and infection, SP-A2 > SP-A1 ≈ KO in male mice, and SP-A2 > SP-A1 ≈ KO (decrease) in female mice. These observations indicate genotype and sex differences in the BAL proteome under the studied conditions. A greater number of proteins were found with altered levels in SP-A2 male mice compared to SP-A1 and this was independent of the presence or absence of ozone exposure. This is consistent with *in vitro* observations, where SP-A2 variants had a higher ability than SP-A1 variants to stimulate cytokine production by macrophage-like THP-1 cells (27, 30, 38), to enhance phagocytosis of bacteria by macrophages (29, 31–33), and to interact with LPS and phospholipids (26, 35). Female mice, on the other hand, showed no differences between SP-A2 and SP-A1 mice under the same conditions. However, if in addition to infection, these were exposed to ozone, the SP-A2 mice had a higher number of proteins with altered levels compared to SP-A1 or KO mice, with no major differences observed between SP-A1 and KO mice in either males or females. This general observation is in line with recent findings showing that the alveolar macrophage miRNome from male SP-A2 mice in response to ozone-induced oxidative stress, differs significantly from that of the SP-A1 (42).

*In vitro* ozone exposure of SP-A variants has been shown previously to decrease the functional activity of SP-A1 and SP-A2 variants (28, 30, 33, 38). Although ozone exposure has been shown to affect the functional activity of SP-A2 relatively more compared to SP-A1, at low physiological concentrations no difference was observed in SP-A2 and SP-A1function after ozone exposure (33) indicating a concentration-dependent effect of ozone-induced oxidation. Whether the differences in the proteomic profiles between hTG SP-A1 and SP-A2 mice may reflect regulatory differences in the translation between SP-A1 and SP-A2 (66) and/or qualitative functional differences between SP-A1 and SP-A2 remains to be determined. For example, there are 15 DEF proteins with marked increase in ozone-exposed hTG SP-A2 male mice compared to ozone-exposed SP-A1 male mice (Table 1S). These proteins could play a critical role in the host defense and inflammatory regulation in the lung in the presence of pneumonia and/or oxidative stress.

Increased levels of proteins related to APR signaling pathway were found in the FA-exposed SP-A1 and SP-A2 mice (except for SP-A2 female where it may decrease) compared to the FA-exposed KO mice; SP-A2 males showed an increase compared to SP-A1 males with no differences between SP-A1 and SP-A2 females. After ozone exposure, SP-A1 and SP-A2 male mice exhibited increased levels of APR related proteins compared to KO male mice, and SP-A2 male mice showed increased APR protein level compared to SP-A1 male mice. Recently as noted above, ozone exposure was shown to differentially affect the microRNAome of the alveolar macrophage in SP-A1 and SP-A2 mice, with SP-A2 mice exhibiting a more robust response (42). However, after ozone exposure, SP-A1 and SP-A2 female mice showed decreased levels of proteins related to the APR signal pathways compared to KO female mice; SP-A2 females showed decreased levels when compared to SP-A1 female mice. Together, these indicate that the BAL proteome is SP-A genotype- and sex-dependent in response to environmental insults.

The APR (ERK1/2 and NF-IL6) signal pathway has been shown to play a critical role in host defense against various infections and in the regulation of inflammation, as well as in oxidative stress (67). The ERK1/2 signaling pathway was involved in an SP-A-enhanced response of macrophages to mycobacteria (68). In the present study SP-A2 and SP-A1 mice compared with SP-A KO mice showed a regulatory effect on BAL protein expression that are associated with the activation of APR (ERK1/2 and NF-IL6) signaling pathways. After ozone exposure, however, only SP-A1 and SP-A2 male mice, but not females, could maintain this regulatory ability (Figure 9). This is of interest and consistent with survival studies where females showed decreased survival after O_3_ exposure of infected mice (13, 14, 69, 70), allowing us to speculate that the regulatory changes observed in males are positive with regards to survival. The present findings indicate that male mice may have a dominant effect or greater ability to regulate the APR (ERK1/2 and NF-IL6) signaling pathway that does not occur in female mice. It is likely that other sex-related factors such as sex hormones are involved in the regulation of the APR (ERK1/2 and NF-IL6) signaling pathway in the response to infection and ozone-induced oxidative stress (14, 42, 43, 71). NF-kB signaling is a critical part of the APR pathway and a few proteins were associated with the NF-kB pathway from the 36 proteins identified in this study. NF-kB signaling is essential for bacterial infection and inflammatory regulation. Previous studies have provided evidence that SP-A activates NF-kB (72) through accumulation of inhibitory IkB-a (73, 74) or direct interaction with cell receptors like TLR-2 and TLR-4 (72, 75–77) or SIRP alpha and CD91 (78). In response to ozone the ability of SP-A to activate NF-kB is decreased as assessed by the lack of changes in the nuclear p65 and cytoplasmic IkB-a levels (79). Furthermore, the data from this *in vivo* study are consistent with the results in WT mice exposed to ozone (14, 50, 56), as well as *in vitro* findings (33, 38), where ozone-exposed SP-A1 and SP-A2 mice reduced (for male) or abrogated (for female) the ability to enhance APR (ERK1/2 and NF-IL6) signaling pathway activation.

Four other signaling pathways including the Nrf2 signaling pathway appeared significant but had fewer proteins than the APR. The Nrf2 signaling pathway has been shown to be a cellular defense mechanism against oxidative stress and inflammation (80, 81). Under normal conditions, Nrf2 is sequestered in the cytoplasm via binding to its repressor molecule Keap1, but under oxidative ER stress, the Nrf2 is released from its repressor and translocated into nucleus to regulate antioxidant molecules. Experimental evidence has shown that the Nrf2 signaling pathway is important for oxidative stress and inflammation (80–82), and Nrf2 signal is a critical regulator of innate immune response and survival during sepsis (83, 84). Nrf2-deficient mice exhibit enhanced susceptibility to severe airway inflammation and asthma (85) and to cigarette smoke-induced emphysema (86). Nrf2 has been shown to be an important pathway in a rescue experiment where SP-A KO mice were treated with exogenous SP-A and the proteomes of the alveolar macrophages from the KO and rescue mice were studied (59, 61). The findings from this study indicate that SP-A1 and SP-A2 infected mice exhibit higher levels of Nrf2-related protein expression than KO mice, suggesting that SP-A plays protective roles against infection and oxidative stress. SP-A1 mice exhibit sex differences in this regard. After ozone exposure the effect of SP-A1 was eliminated, but SP-A2 males (but not females) still showed increased level of proteins associated with the Nrf2 pathway (Figure 9). This is consistent with recent findings where the SP-A2 male miRNome of the alveolar macrophage exhibited a robust response after ozone exposure compared to SP-A1 males and females and SP-A2 females (42). Furthermore, male mice after ozone exposure and infection had been shown to have a better survival compared to females (13, 14, 52) and these sex differences were minimized or eliminated after gonadectomy (71) implicating sex hormones. Therefore, it is possible that sex hormones play a role in this SP-A1/SP-A2 genotype-sex interaction.

Of interest, the Nrf2 pathway is part of the xenobiotic metabolism signaling pathway, which was also associated with protein changes in this study. In addition, the glutathione metabolism pathway and the aryl hydrocarbon receptor signaling pathway are associated with the protein changes in this model. These pathways share three proteins, i.e., glutathione S-transferase A1, glutathione S transferase, omega 1, and glutathione S-transferase A4, which are present in the Nrf2 and the xenobiotic metabolism signaling pathways. The major function of the glutathinone S-transferase superfamily is to detoxify xenobiotics by catalyzing the nucleophilic attack by GSH on electrophilic carbon, sulfur, or nitrogen atoms of nonpolar xenobiotic substrates, thus preventing the damage of crucial cellular proteins and nucleic acids in cells (87).

Eleven proteins were observed to be statistically significant different among mice with different SP-A genotype and with or without ozone exposure. SP-A2 mice showed higher levels of annexin A5, SP-A, 14-3-3 zeta protein, and glutathione S-transferase A4 than SP-A1 and KO mice in response to ozone and infection (Figure 4). These proteins are involved in host defense, immunity functions, and redox balance. Therefore, higher levels of defense and immunity proteins are likely to enhance the ability of SP-A2 mice to protect the lung from bacterial infection and ozone-induced oxidative stress. Annexin A5 regulates the permeability of the channel pore to ions and plays a central role in the machinery of membrane repair (88, 89). In addition, 14-3-3 zeta protein is an important regulator for a wide range of regulatory processes, such as mitogenic signal transduction, and apoptotic cell death (64). Although members of the 14-3-3 family regulate SP-A2 expression by directly binding at the 5′UTR, the 14-3-3 zeta isoform did not appear to be involved (66). In infected SP-A2 mice the 14-3-3 zeta protein level was higher than in infected SP-A1 and KO mice (Figure 4C). Whether this higher level could improve neutrophil cell apoptosis and benefit apoptotic cell clearance in the alveolar space remains to be determined. Moreover, as mentioned above, infected SP-A2 mice could increase the expression levels of the Nrf2 signaling pathway-related proteins, such as members of glutathione S-transferase family. The higher levels of these Nrf2-related proteins may enhance the defense ability in hTG SP-A2 mice against various infection and environmental insults such as oxidative stress and LPS exposure (80, 85, 90). Although SP-A1 mice have a lower level in host defense-related protein expression compared to SP-A2 mice, about one half of the 36 identified proteins in SP-A1 mice increased when compared to KO mice after infection. However, these increases were abrogated after ozone exposure. This indicates that the biological function mediated by these proteins in hTG mice could be significantly impaired *in vivo* by ozone-induced oxidation. In fact, SP-A oxidation in WT mice after ozone exposure has been observed (52, 53) and this may result in a reduction of function (52).

In summary, the findings of the present study, where the *in vivo* differential effects of SP-A genotypes were investigated on the BAL proteomic patterns and associated signaling pathways in response to ozone or FA exposure and bacterial infection, revealed: (a) ozone exposure significantly increases the level of total BAL proteins compared to bacterial infection alone; (b) of the 36 proteins identified in BAL fluid, 11 were found with significant differences in one or more comparisons of the study groups; (c) APR and Nrf2 related signaling pathways were identified as important pathways in response to SP-A genotype, sex and ozone/FA exposure in this bacterial infection. The NF-kB signaling, a critical part of the APR pathway may also play an important role. These findings may provide new insight into molecular mechanisms of human SP-A1 and SP-A2 genetic variants in response to environmental insults.

## Author Contributions

GW, SH, and AM performed animal experiments. GW and TU carried out data analysis and result interpretation. DP guided proteomic analysis and result interpretation. GW and JF designed the experiments and wrote the manuscript. JF provided oversight throughout this project.

### Conflict of Interest Statement

The authors declare that the research was conducted in the absence of any commercial or financial relationships that could be construed as a potential conflict of interest.
