# Supplementary material for: Differential Effects of Human SP-A1 and SP-A2 on the BAL Proteome and Signaling Pathways in Response to Klebsiella pneumoniae and Ozone Exposure
Source: Front Immunol. 2019 Mar 26;10:561. doi: 10.3389/fimmu.2019.00561 (PMC6443908; doi:10.3389/fimmu.2019.00561)
Supplement: Supplementary file 5 [file Data_Sheet_2.docx]

Supplemental File 2

MIAPE: Gel Informatics

*Version 1.0, July, 2009*

Reporting requirements for gel informatics data

1. General features
   1. Date stamp: 2009-09-19
   2. Responsible person or institutional role: Todd M. Umstead, Senior Research Support Associate, Penn State Center for Host defense, Inflammation, and Lung Disease (CHILD) Research, Department of Pediatrics, P.O. Box 850, Hershey, PA 17033
   3. Electrophoresis type: 2D-DIGE with PAGE electrophoresis
   4. Electrophoresis context: Proteomic analysis of bronchoalveolar lavage (BAL) proteins from SP-A knockout (KO) male and female mice and humanized SFTPA1 and SFTPA2 transgenic male and female mice after *Klebsiella pneumoniae* infection with or without ozone exposure
   5. Image(s): Available upon request
   6. Image analysis software: Progenesis SameSpots v3.0 (Nonlinear Dynamics)
   7. Statistical analysis software: Progenesis SameSpots v3.0 (Nonlinear Dynamics), Excel (Microsoft), SigmaStat (SPSS)
2. Gel analysis design
   1. Type: Directed
   2. Replicates: n=4 per group
   3. Groups: 12 groups (SP-A knockout males exposed to filtered air, SP-A knockout males exposed to ozone, SFTPA1 transgenic males exposed to filtered air, SFTPA1 transgenic males exposed to ozone, SFTPA2 transgenic males exposed to filtered air, SFTPA2 transgenic males exposed to ozone, SP-A knockout females exposed to filtered air, SP-A knockout females exposed to ozone, SFTPA1 transgenic females exposed to filtered air, SFTPA1 transgenic females exposed to ozone, SFTPA2 transgenic females exposed to filtered air, and SFTPA2 transgenic females exposed to ozone) (all groups were infected with Klebsiella pneumoniae after exposure to filtered air or ozone)
   4. Internal standard: Cy2 normalization pool of equal amount of protein from all study samples run on each analytical gel
   5. External standard: Cy3/Cy5 counterbalancing to eliminate dye-based artifacts
3. Image preparation
   1. Software: ImageQuant TL (GE)
   2. Preparation steps: Images obtained using the Typhoon 9400 Variable Mode Imager (GE Healthcare) in the GEL file format (.gel) and cropped using ImageQuant TL (GE)
      1. Analytical (quantitative) gels: Laser voltages were optimized for each fluorophore prior to scanning to avoid signal saturation. Identical laser settings were then used to scan each gel
      2. Preparative/picking gels: Fixed with ethanol/acetic acid and post-stained with Deep Purple Total Protein Stain (GE), scanned independently from analytical gels
      3. All gels were scanned at 100μm resolution
      4. See MIAPE Gel Electrophoresis supplement for more specific details of image collection
4. Image analysis pre-processing
   1. Input image(s): Images obtained using the Typhoon 9400 Variable Mode Imager (GE) in the GEL file format (.gel) and cropped using ImageQuant TL (GE)
   2. Software***:*** ImageQuant TL (GE)
   3. Processing steps: See image preparation above
5. Data extraction process
   1. Input image(s): Images obtained using the Typhoon 9400 Variable Mode Imager (GE) in the GEL file format (.gel) and cropped using ImageQuant TL (GE) and are available upon request
   2. Image quality control: Image QC done using Progenesis SameSpots v3.0 (Nonlinear Dynamics) to check images for bit depth, color, manipulation prior to analysis, proper file type, saturation, low dynamic range, and stretched contrast
   3. Image alignment
      1. Automatic gel alignment using Progenesis SameSpots v3.0 (Nonlinear Dynamics) to allow for more accurate spot matching
      2. Gel alignment manually edited following automated alignment protocols
   4. Feature detection
      1. Automatic spot detection using Progenesis SameSpots v3.0 (Nonlinear Dynamics)
      2. Features were manually edited following automated spot detection protocols
   5. Matching
      1. Algorithm: Progenesis SameSpots v3.0 (Nonlinear Dynamics)
      2. Reference image(s) used: 79457 Standard Cy2 aligned
      3. Landmarks: Vectors were automatically and manually placed
      4. Match editing: Automatic spot matching using Progenesis SameSpots v3.0 (Nonlinear Dynamics) followed by manual edited to confirm matches
      5. One-hundred percent spot matching across all gels without missing values was set as a requirement for spot inclusion for data analysis
   6. Feature quantitation
      1. Type: Normalized Volume
      2. Quantitation: Progenesis SameSpots v3.0 (Nonlinear Dynamics)
      3. Background subtraction: N/A
      4. Normalization: Progenesis SameSpots v3.0 (Nonlinear Dynamics)
6. Data analysis
   1. Analysis intent: Features with ANOVA (p<0.05) and/or t-test (p<0.05)
   2. Software: Progenesis SameSpots v3.0 (Nonlinear Dynamics), Excel (Microsoft), SigmaStat (SPSS)
   3. Type: ANOVA with false-discovery rates based on Progenesis assigned q-value, t-test, principal component analysis (PCA)
   4. Parameters: Not blinded
   5. Input data: Normalized volume (Cy3/Cy2 and Cy5/Cy2)
7. Data reporting
   1. List of image features: Excel file available upon request
   2. List of matches: Excel file available upon request
   3. Description of analysis results: Excel file available upon request
